# 

**Published:** 1879-01-20

**Authors:** 


Abdominal affections, clinical value of the thermometer in ... 188
Abdominal typhus, contagiousness of ... 350
Abingdon academy of medicine ... 238
Aborted croupus pneumonia ... 401
Abortifacient, pilocarpin as an ... 130
Abortion, management of retained secundines in ... 415
Abortion, viburnum prunifolium for controlling the habit to ... 106
Abortion, viburnum prunif. in threatened ... 47
Abrasions, ulcers and ... 436
Abscess, arrest of milk ... 140
Abcess, mammary ... 150 394
Accidents from using anæsthetics, prevention of ... 226
Acetate of potass. in pneumonia and rheumatic fever ... 109
Acne, pimply face ... 97
Aconite ... 155
Aconite in pneumonia ... 404
Action of idioform ... 228
Action of quinine, to hasten ... 350
Active principles of squill ... 385
Acute glaucoma in a woman 49 years old ... 270
Acute nephritis ... 465
Acute testitis, remedial management of ... 436
Address, introductory, upon southern institutions ... 441
Affection, dropsical ... 394
Affections, bismuth in skin ... 225
After delivery ... 423
After-pains ... 73
Air purifier ... 192
Albumen occurs, when ... 390
Albumen, test for ... 316
Albuminuria of pregnancy, Lithia waters for ... 255
Albuminuria, pilocarpin in ... 108
Albuminuria, treatment of ... 105
Albuminuria treated by oxygen ... 430
Alcoholism, treatment of ... 138
Alcohol in fever ... 409
Aleurites, oil of triloba as a cathartic ... 100
Alexis St. Martin ... 431
Alkalies in dyspepsia ... 188
Alkaline columbo draught ... 193
Alkáloid, cinchonia ... 153
Alkaloids of opium ... 58
Alopecia or baldness ... 75
Alpha ... 232
Alterative pills, pulmonary ... 74
Amenorrhœa, a case of ... 467
Amenorrhœa, apiol in ... 310
Amenorrhœa, electricity in ... 462
American medical association ... 157
Ammoniacal sulphate of copper in neuralgia of the fifth nerve ... 416
Ammonia, picrate of ... 101
Amnii, liquor ... 464
Amorphia as an expectorant ... 180
Amyl, nitrite of ... 18
Anæsthesia, discoverer of ... 310
Anæsthetic ... 192
Anæsthetic, dichloride of ethidene as an ... 227
Anæsthetic for children ... 256
Anæsthetics, prevention of accidents from ... 226
Anatherine dentitrice, Popp's ... 435
Aneurism of the aorta ... 19
Angina, gargle for ... 235
Ani, pruritus ... 418
Ano, fistula in ... 89, 145
Anorexia ... 98
Anteflexion of the uterus ... 433
Anteflexion of the womb ... 105
Anthrax, cupping in ... 176
Anti-asthmatic powder ... 475
Anticipation of the microphone ... 4[illegible]2
Antidote for arsenite of soda ... 2[illegible]
Antidote to bad air ... 28
Antidote to arsenic ... 225
Antidote to poison ... 265
Anti-emetic ... 109
Anti-emetic, peach leaves as ... 76
Anti-emetics ... 34, 36
Antipyretic ... 66
Antipyretic, bromo-hydrate of quinine as ... 73, 225
Antipyretic, salicylate of quinine as an ... 220
Antiscorbutic, salicylic acid as an ... 396
Antiseptic ... 66
Antiseptic dressing, experiments with ... 61
Antiseptic gauze ... 63
Ants, division of labor among ... 472
Aorta, aneurism of the ... 19
Aorta, diagnosis of aneurism in ... 69
Aperient, useful ... 195
Apiol ... 63
Apiol in amenorrhœa and dysmenorrhœa ... 310
Apomorphia, nouvel use for ... 148
Appearance of the tongue in diseases ... 378
Apoplexy, treatment of cerebral ... 103
Arenaria rubra in diseases of the bladder ... 414
Arrowroot for infants ... 269
Arsenic, antidote to ... 225
Arsenic as a blood and cardiac tonic ... 266
Arsenic, delicate test for ... 72
Arsenite of soda, antidote to ... 226
Artificial cataplasm ... 353
Artificial respiration ... 18
Ascarides, to expel ... 355
Asclepias in scrofula ... 114
Aspirator, a simple ... 141
Aspirator in hernia ... 384
Asthma, treatment of ... 269
Astronomical observations ... 192
Atmosphere, wonders of ... 112
Atropia and datura not identical ... 68
Atropia in croup ... 466

B

Bad air, effectual antidote to ... 28
Baldness or alopecia ... 75
Baldness, treatment of ... 102
Bandage, Martin's, in chronic ulcer ... 381
Baptisia tinctoria in typhoid fever ... 306
Barber's itch ... 435
Baths, turpentine vapor ... 67
Battey's operation ... 147
Bedford alum and iron springs ... 129
Beef, Colden's Liebig's liquid extract of ... 211
Beef juice and ice cream ... 469
Belladonna hypodermically in congestive chills ... 230
Benzoated cotton-wool ... 69
Benzoate of Lithia ... 101
Benzoic acid in articular rheumatism ... 68
Bismuth in skin affections ... 225
Bismuth, liquor, for nasal catarrh ... 106
Bismuth, subnitrate of ... 30, 67
Bladder, inflammation of the ... 27
Bladder, stigmata of maize and arenaria rubra in diseases of the ... 414
Blasting agent, a new ... 72
Bleeding hemorrhoids ... 73
Blennostatic properties, kava-kava and its ... 345
Blepharitis ... 69, 121
Blepharitis, vulcanized india rubber in ... 108
Blisters prevented, strangury from ... 266
Blister, treatment of carbuncle by ... 226
Blood tonic, arsenic as a ... 266
Bodies on the iris, foreign ... 453
Boldo, a stimulant to digestion ... 468
Boils, to prevent ... 107
Book notices ... 40, 80, 119, 238, 277, 318, 357, 398, 439, 479

Boracic acid ointment.... 15[illegible]
Borax, physiological action of.... 272
Breasts, gathered.... 459
Brain. wounds to the.... 366
Brandy, hypodermic injection of.... 142
Bread, laxative.... 395
Breath, remedy for foul.... 233
Breech presentations.... 227
Bright's disease, chronic.... 64, 350
Bright's disease, danger of opium in chronic... 109
Bromide of ammonium in too frequent menstruation.... 270
Bromide of potash in cholera infantum.... 235
Bromide of potassium, external application of 386
Bromide of potassium in cough.... 190
Bromine injections in cancer of the womb.... 270
Bromo-hydrate of quinine as an antipyretic... 73
Bronchi mistura.... 236
Bronchitis.... 114
Bronchitis, eucalyptus in.... 470
Bronchitis from mouth breathing.... 218
Bronchitis ,vinegar fumes in.... 66
Brooklyn anatomical and surgical club.... 248
Brunella vulgaris.... 59
Bryonia and drosera in whooping cough... 110, 266
Bryony, therapeutic effects of.... 268
Buckeye, infusion of, as as a remedy for chronic rheumatism.... 469
Buffalo Lithia water for uræmia, etc.... 255
Burns,.... 74, 396
Burns, charcoal for.... 68
Burns, treatment of.... 154, 189

C

Cachexia.... 195
Cactus and heart's ease.... 30
Cæcum and colon, cases of impacted.... 449
Cæsarean section.... 411
California remedies, new.... 77
Calomel.... 393
Calomel as an anti-emetic.... 34
Caloptine.... 152
Camera obscura.... 231
Camphorated Dover's powder.... 475
Cancer, Esmarch's treatment of.... 76
Cancer, guaco in.... 190
Cancer of the cervix.... 457
Cancer of the womb treated by bromine injections.... 270
Cancer of stomach, codeia for.... 70
Cancer, removal of rectum for the relief of.... 19
Cancer treated by eucalyptus.... 333
Capsicum with quinia.... 195
Carbolic acid as an anti-emetic.... 34
Carbolic acid in erysipelas.... 150
Carbolic acid, injecting pile tumors with.... 30
Carbolic acid in nasal polypus.... 229
Carbolic acid in surgical diseases, use of.... 28
Carbolic acid poisoning.... 29, 389
Carbolic acid spray in coughs.... 102
Carbolic acid spray in diphtheria.... 69
Carbolized jute as a wound dressing.... 174
Carbonic oxyde by living organisms, absorption of.... 152
Carbuncle of the urethra.... 457
Carbuncle, treatment with blister.... 226
Cardiac dyspnœa.... 356
Cardiac dyspnœa, treatment of.... 424
Cardiac tonic, arsenic as a.... 266
Carya oliviformis.... 36
Cascara sagrado.... 36
Castor oil a dressing for leather.... 236
Castor oil palatable.... 313
Cataplasm, artificial.... 353
Cataract.... 230
Cataract extraction in an unpromising case.... 270
Cataract, extraction of.... 303
Catarrhal affections, yerba santa in.... 476
Catarrhal fever.... 116, 117
Catarrh, treatment of.... 91
Catgut ligature.... 19
Cathartic, an effective.... 428
Cathartic, oil of aleurites triloba as a.... 100
Catarrh, post nasal.... 434, 474
Catheterism, female.... 183
Catheter, Jacques'.... 30
Cause of diarrhœa, dentition as a.... 390
Cause of thunder.... 391
Caustics, use of certain.... 51
Cephalic version by external manipulation.... 168
Cerebral apoplexy, treatment of.... 103
Cerebral hemorrhage, prognosis in.... 100
Cervix, cancer of the.... 457
Cervix, dilating the.... 138
Cervix uteri, plugging the.... 464
Chancre, perchloride of iron as a topical application for.... 35
Chancroids, hot water in.... 428
Charcoal for burns.... 68
Chaulmoogra oil.... 354
Childbirth without pain.... 86
Child, fever in a.... 433
Children, anæsthetic for.... 256
Children, diarrhœa of.... 316
Children, enuresis in.... 355
Children, irritable stomach of.... 195
Children, incontinence of urine in.... 388
Chloral, application of.... 147
Chloral as a counter-irritant.... 194
Chloral, enemata of.... 27
Chloral hydrate enemata.... 56
Chloral hydrate in obstetrics.... 88
Chloral in convulsions, hypoderm. inject. of... 387
Chloral in dysentery.... 231
Chloral in puerperal eclampsia.... 94
Chloral in retention of urine.... 225
Chloral, syrup of.... 75
Chlorine mixture in scarl. fever and diphther. 185
Chloroform in convulsions of infants and children.... 165
Chloroform in diseases of the heart.... 148
Chloroform in labor.... 217
Chloroform, tetanus and.... 110
Choice of purgatives.... 389
Cholalogues.... 387
Cholera, coffee in.... 349
Cholera, dysentery in.... 234
Cholera, hog.... 315
Cholera infantum.... 69, 393
Cholera infantum, bromide of potash in.... 235
Cholera infantum, dysentery and.... 234
Cholera infantum, monobromated camph. in.. 236
Cholera infantum, pepsin and creosote in.... 234
Cholera infantum, subnitrate of bismuth in.... 234
Cholera, jaborandi in.... 382
Cholera infantum, tinct. of iodine in.... 276
Cholera morbus.... 396
Cholera morbus, hypodermic medication in... 335
Cholera, paracotoin in epidemic.... 150
Cholera, sulphuric acid in.... 475
Chronic bronchitis.... 194
Chronic Bright's disease.... 350
Chronic ulcers, Martin's bandage in.... 381
Cinchonia, sulphate of.... 7
Cinchonia alkaloid.... 207
Cinchonidia, urticaria caused by sulphate.... 147
Civilization, longevity and.... 232
Clearing water, peach kernels for.... 310
Clocks, self-luminous.... 152
Codeia as a sedative.... 297
Codeia for cancer of stomach.... 70
Coffee in cholera.... 349
Cold, application of, and elevation of an extremity as hemostatics.... 184
Colden's Liebig's liquid extract of beef.... 211
College museum and library.... 398
Colliquative diarrhœa.... 109
Collodion, Paresi's hemostatic.... 76
Concentrated solution of salicylic acid.... 354
Conflict of opinion.... 469
Congenital syphilis.... 459
Congestive chills, belladonna hypodermic. in 230
Constipation, habitual.... 460
Consumption, cough mixture for.... 194
Consumption, is it contagious?.... 1
Consumptives to travel, rules for sending.... 98
Constipation, glycerine as an enema in.... 146
Constipation, habitual.... 153
Constipation, cure of habitual.... 382
Contagiousness in consumption.... 1
Contagiousness of abdominal typhus.... 350
Contagium of syphilis.... 349
Convention of medical colleges.... 237

Convulsions, hypodermic inject. of chloral in.. 387
Convulsions, jaborandi in puerperal... 97
Convulsions of infants, chloroform in... 165
Convulsions, periodical... 156
Convulsions, puerperal... 30
Convulsions, prophylaxis of puerperal... 212
Coryza... 235
Coryza, cure of... 395
Cosmeticum, Henry's... 116
Cost of yellow fever... 192
Cotton-wool, benzoated... 69
Cough, how to... 101
Cough mixture... 75
Cough-mixture for consumption... 194
Cough, oxalate of cerium in... 59
Cough, pathology of hysterical... 139
Coughs, bromide of potassium in... 190
Counter-irritant, chloral as a... 194
Cornea, phlyctenular ulcer of the... 307
Cornea to the human eye, transplantation of a dog's... 347
Cracked nipples... 105
Crater in the moon, new... 191
Crawford W. Long, honor to... 349
Creosote as an anti-emetic... 36
Creosote in cholera infantum... 234
Croton chloral, therapeutic value of... 412
Croton oil, treatment of nævus by... 146
Croup, atropia in... 466
Croupous pneumonia... 401
Croupous pneumonia, a specific infectious disease... 201, 237
Cubebs in whooping cough... 476
Cucumber, tape worm in... 68
Cupping in anthrax... 176
Cure for hiccough... 190
Curvatures, spinal... 216
Cyst, diagnosis of the ovarian... 460
Cystitis, milk diet in... 98

D

Dacrocystitis... 122
Dangers of vulcanized rubber nipples... 236
Daturia and atropia not identical... 68
Deaf hear, making the... 392
Death, a forerunner of... 224
Death in uterine injections, cause of... 348
Delivery, post mortem... 137
Dentition as a cause of diarrhœa... 390
Dextro-quinine in pertussis... 417
Diabetes... 33
Diagnosis, differential, between true epilepsy and hystero-epilepsy... 229
Diagnosis in aneurism of aorta... 69
Diagnosis of gastric disorders, condition of the tongue valuable in... 229
Diagnosis of the ovarian cyst... 460
Dialysed iron... 17
Diaphragm, rheumatism of the... 221
Diarrhœa, colliquative... 109
Diarrhœa, dentition as a cause of... 390
Diarrhœa in infant from improper feeding... 128
Diarrhœa of children... 316
Diarrhœa, oxide of zinc in... 223
Diarrhœa, painful... 394
Diathesis, uric acid... 109
Dichloride of ethidene as an anesthetic... 227
Dietetics, moral... 431, 466
Diet, milk... 110
Differential diagnosis between true epilepsy and hystero-epilepsy... 229
Diffusion of gases... 471, 472
Digitalis, external use of, in suppress. of urine. 190
Dilating the cervix... 138
Diphtheria... 70, 149, 154, 209
Diphtheria, chlorine mixture in... 185
Diphtheria, carbolic acid spray in... 69
Diphtheria, incipient... 29
Diphtheria, potato eating and... 187, 315
Diphtheria, pycrate of ammonia in... 101
Diphtheria, salicylic acid in... 33
Diphtheria, the continuity theory of... 281
Diphtheria, treatment of... 184
Discoverer of anesthesia... 210
Disinfectant, cheap... 267
Disinfectant, sanitas,, the new... 468
Disinfectant, self-generating... 105
Distemper, treatment of... 104
Dog fennel... 156
Division of labor among ants... 472
Dolbeur's experiments... 32
Dover's powder, camphorated... 475
Dover's powder, modified... 356
Dreaming, rapidity of thought in... 231
Dressing, experiments with antiseptic... 61
Dressing, head wounds... 316, 376
Dressings in wounds... 108
Dropsical affection... 394
Drops, toothache... 34
Dropsy, treatment of... 474
Drosera in whooping cough... 110, 266
Drowned, treatment of the... 447
Drowning, resuscitation of a young girl apparently dead from... 290
Duty on quinine... 438
Dysentery and cholera infantum... 234
Dysentery, belladonna in... 234
Dysentery, chloral in... 234
Dysentery, foul water and... 346
Dysentery, injections of water in... 465
Dysentery treated by injections of a solution of chloral... 466
Dysentery, remedy for... 222
Dysentery, salicylic acid enemata in... 190, 268
Dysentery, sulphate of magnesia... 234
Dysmenorrhœa... 35
Dysmenorrhœa, apiol in... 310
Dysmenorrhœa, membranous... 469
Dyspepsia... 253, 275
Dyspepsia, alkalies in... 188
Dyspepsia, prescription for... 74
Dyspepsia, relief for... 35
Dyspnœa, cardiac... 356

E

Ear disease, salicylic acid in... 394
Ear, foreign body in... 9
Ear, polypi of the... 67
Earth quake, how deeply does the... 311
Eclampsia, chloral in... 94
Eclampsia, puerperal... 262
Eclampsia, when to induce premature labor in 384
Eczema of the hand, treatment of... 334
Electric baths, how to give... 104
Electric gas lighting apparatus... 191
Electricity, a new theory of... 232
Electricity for sleeplessness... 65
Electricity in amenorrhœa... 452
Electricity in impotence... 463
Electricity in nervous vomiting... 429
Electric light... 351, 392
Electro therapeutics... 373
Elephantiasis Græcorum... 126
Elephant, pregnancy in... 151
Elixir simple... 275
Empyema or suppurative pleuritis... 286
Emulsio expectorans... 236
Enemata, chloral hydrate... 56
Enemata of chloral... 27
Enlarged glands in scarlet fever... 149
Enlargement of the right tesiis in child two years of age... 403
Enuresis in children... 355
Enuresis nocturna... 100
Epididymitis, improvement in treatment of... 172
Epilepsy... 149
Epilepsy and hystero-epilepsy, differential diagnosis between... 229
Epilepsy, ergot in... 228
Epilepsy, sodium bromide in... 228
Epileptic attack, means of arresting... 456
Epistaxis, ergotic hypodermics in... 316
Epithelioma... 223
Ergotic hypodermics in epistaxis... 316
Ergot in epilepsy... 228
Ergotin, hypodermic injection of... 276
Ergotin in acute ophthalmia... 144
Ergotin in neuralgia, injection of... 388
Ergotin, injections of, in cerebral apoplexy... 103
Ergotin in ophthalmia... 230
Ergotin suppositories... 115
Ergot, topical use of... 354

Ergot *vs.* lactation 310
Erysipelas, injections of carbolic acid in 150
Esmarch's tourniquet in neuralgia 386
Esmarch's treatment of cancer 76
Ether, cure of sciatica by hypod. injection of 49
Ether, first insensibility from 470
Ether, insensibility from inhalation of 222
Ethylate of sodium 348
Eucalyptus cancer treated by 333
Eucalyptus in bronchitis 470
Eucalyptus oil, inhalation of 355
Euonymus atropurpureus 258
Excoriated nipples, suberine in 267
Exophthalmic goitre 90
Expectorant, amorphia as an 180
Experiments, Dolbeur's 32
Experiments, Prof. Jones' with the water and air from the yellow fever rooms 181
Experiments to illustrate geological process 151
Explosive mixture 309
External application of the bromide of potass. 386
Extraction of cataract 303
Extract of beef, Colden's Liebig's liquid 211
Extravasation, bloodletting in 123
Eye, injurious effect of tobacco on the 64
Eye-sight, tobacco poisoning and its effects on the 321

F

Facetiæ 310
Fancher case 118
Fecundity and sexuality 352
Feet, cold, causing sleeplesness 67
Feet, frosted 76
Female catheterism 183
Female, cystitis in the 458
Feted discharges from tooth abscess opening into the anthrum 395
Fever, alcohol in 409
Fever, Berlin treatment of scarlet 146
Fever, catarrhal 116, 117
Fever in a child 433
Fever, incubative period of scarlet 26
Fever, intermittent 276
Fever, Prussian blue in 141
Fever puerperal 458
Fibroid tumor of the uterus treated by sclerotic acid 70
Fish-bones in the pharynx 224
Fissure of the rectum 460
Fistula in ano [illegible]89, 145
Flatulent dyspepsia 65
Floral guide, Vick's 39
Fomentations, warm, to the head in uterine hemorrhage 148
Food, glycerine as a 342
Foreign body in the ear 9
Forerunner of death 224
Formation of whirlwind 72
Foul breath, remedy for 233
Foul water and dysentery 346
Freckles 194
Frosted feet and hands 76
Frost, effects of 112
Fuchsine in albuminuria 105
Funis, treatment of the 459

G

Gallium 232
Galvanic current in exophthalmic goitre 377
Galvanism, medicine by 351
Galvanism, opium habit cured by 332
Gargle for angina 235
Gases, diffusion of 471, 472
Gas intestinal 65
Gastric disorders, condition of the tongue valuable in the diagnosis of 229
Gathered breasts 459
Gelsemium for hectic 186
Gentian and rhubarb draught 193
Geological process, experiment to illustrate 151
Gingivitis in pregnancy 66
Gingivitis of puerperal women 429
Glanders, poisoning by 265
Gleet, treatment of 107
Globe, the human population of the 112
Glycerin as a food 342
Glycerin as an enema in constipation 146
Glycerine in phthisis 430
Glycerole of thymol 435
Gnoscopine 432
Goitre, exophthalmic 90
Goitre, galvanic current in 377
Gonorrheal ophthalmia 123
Gonorrhœa 75, 374, 395
Gonorrhœa, how to cure it 290
Gonorrhœa, injection for 115
Gonorrhœa, treatment of 70, 356
Gossamers 232
Graafian vesicle during pregnancy 107
Grafting, modus operandi of 21
Green-eyed phantom 478
Guaco in cancer 190
Gunpowder, sanitary uses of 112

H

Habit, opium 269, 270
Hæmostatics, application of cold as 184
Hair-dies 74
Hair-dye without lead or silver 193
Hairy water tortoise 152
Hands, chapped 115
Hands, frosted feet and 76
Hawkweed 75
Hay fever, remedy for 345
Headache, enemata of chloral in sick 27
Headache, hints for 189
Head, injuries of the 123
Head wounds, dressing 316, 376
Heart, chloral in diseases of the 148
Heart, neuroses of the 107
Heart's action, hypodermic use of morphia as a supporter of 325
Heart's-ease 30
Hectic, gelsemium for 186
Hemorrhage from the nose 396
Hemorrhoids, internal, treated by hypodermic medication 455
Hemorrhage, post-partum 105
Hemorrhage, pyrogallic acid in 265
Hemorrhage, to restrain 193
Hemorrhoids, bleeding 73
Hemorrhoids, fatal tetanus following ligation of 146
Hemorrhoids, treatment of 266
Hemostatic collodion, Paresi's 76
Hernia, effect of posture in strangulated and incarcerated 257
Hernia, hypodermic injection in 102
Hernia, the aspirator in 384
Hernia, treatment of 476
Herpes and arsenic 150
Herpes circinnatus or ring worm 73
Herpes zoster 148
Hiccough 228, 236
Hiccough, œsophageal sound in 310
Hiccough, cure for 190
Hiccough, therapentics of 343
Hints for headache 189
Hoax, a scientific 272
Hog cholera 315
Honor to Dr. Crawford W. Long 349
Horrible! 68
Horse, a substitute for 271
Hot water and creosote as an anti-emetic 36
How deeply does the earth quake 311
How old is the world 311
Hydrobromate of quinia as an antipyretic 225
Hydrobromic acid 274
Hypodermic use of morphia 406
Hydroscope and thermoscope 208
Hyoscyamine 145
Hypertrophied tonsils, lemon juice for 350
Hypnotic, new 195
Hypnotic, monobromide of camphor as a 28
Hypochondriasis, sexual 361
Hypodermic injection in hernia 102
Hypodermic injection of brandy 142
Hypodermic inject. of chloral in convulsions 387
Hypodermic injection of ergotin 276
Hypodermic medication in cholera morbus 335

Hypodermic morphia, sudden stoppage of...... 209
Hypophosphites, value of the........ 337
Hysterical cough........ 139

I

Ice cream and beef juice........ 469
Ice cream poisoning........ 24
Ice in the rectum in chloroform narcosis........ 110
Ice, mammary inflammation treated by........ 389
Icteric, uterine........ 110
Immersion, treatment of unhealthy and syphilitic sores by........ 341
Impacted cæcum and colon........ 449
Impotence, electricity in........ 463
Impotency........ 115
Incipient diphtheria........ 29
Incontinence of urine in children........ 388
Incubative period of scarlet fever and other diseases........ 26
Indestructibility of matter........ 471
Index medicus........ 39
Infantile convulsions, nitrite of amyl in........ 106
Infantile paralysis........ 223
Infants, arrowroot for........ 269
Infantum, cholera........ 69, 393
Infancy, typhoid fever during........ 308
Inflammation, mammary, treated by ice........ 389
Inflammation of the bladder........ 27
Infra-orbital nerve, resection of........ 267
Inhalation of ether, insensibility from........ 222
Inhalation of eucalyptus oil........ 355
Injecting a tumor with morphia before extirpation........ 106
Injecting pile tumors with carbolic acid........ 30
Injection for gonorrhœa........ 115
Injections of ergotin in cerebral appoplexy........ 103
Ink, invisible........ 152
Insensibility from ether........ 470
Insensibility from inhalation of ether........ 222
Insensibility, protoxide of nitrogen for producing prolonged........ 294
Insolation........ 292
Insomnia........ 69
Insufflation powders *vs.* nasal douche........ 105
Intermittent fever........ 276
Internal hemorrhoids, treated by hypodermic medication........ 455
Interstitial pneumonia........ 388
Intra-uterine medication........ 463
Intravenous injections........ 340
Intussusception in infants........ 189
Inventions, new........ 71, 72
Inverted toe nail........ 264
Iodide of potassium in vomiting........ 269
Iodoform........ 383
Iodoform, action of........ 228
Ipecac as an anti-emetic........ 34
Ipecac in labor........ 35
Iris, foreign bodies on the........ 453
Iritis, rheumatic........ 394
Iron, dialysed........ 17
Irritable stomach of young children........ 195
It steps down from its airy throne........ 328

J

Jaborandi in cholera........ 383
Jaborandi in puerperal convulsions........ 97
Jaborandi in whooping cough........ 215
Jaborandi, the use of........ 25
Jacques' catheter........ 30

K

Kava-kava and its blennostatic properties........ 345
Kidney worm........ 71
Kolpoecpetasis *vs.* Kolpokleisis........ 476

L

Labor, chloroform in........ 217
Labor in puerperal eclampsia, when to induce 384
Labor, use of ipecac in........ 35
Labor, warm water into the vagina in........ 301
Laceration of the perineum........ 757
Lactation, ergot *vs*........ 310
Lactopeptine........ 75, 218
Laparotomy........ 20
Laryngitis, syphilitic........ 68
Laxative bread........ 395
Laxative mixture, Bossu's........ 116
Laxative tonic pills........ 195
Leather, castor oil a dressing for........ 236
Lemon juice for hypertrophied tonsils........ 350
Leprosy, a case of........ 126
Ligature, catgut........ 19
Light, electric........ 351, 392
Linseed in skin diseases........ 263
Lip salve........ 435
Liquid glue........ 434
Liquor amnii........ 464
Liquor arsenicalis in prickly heat........ 195
Lithia, benzoate of........ 101
Lithia springs, waters of........ 177
Local treatment of diseases of the skin........ 368
Locomotor attaxie progressive........ 5
Longevity and civilization........ 232
Loss of blood, whiskey hypodermically in collapse from........ 164
Lumbago, sulphuric ether in........ 147
Lupus, treatment of........ 96

M

Making the deaf hear........ 392
Malarial fever of the South........ 315
Malarial fever, Prussian blue in........ 141
Malarial fever, to prevent........ 476
Malnutrition and diarrhœa........ 128
Mammary abscess........ 150
Mammary abscess, preventing........ 375, 394
Mammary inflammation treated by ice........ 389
Management of retained secundines in abortion........ 415
Marriage and the microscope........ 143
Mastitis, phytolacca decandra as a remedial agent in........ 408
Massage of the tonsils........ 309
Mastoid process, opening the, by surgical procedure........ 259
Matter, the constitution of, in gaseous state... 111
Medical association, American........ 117, 198
Medical College Association........ 157
Medical College, Southern........ 77
Medical College, the new........ 37
Medical editors, Association of American........ 197
Medical journals, importance of........ 158
Medical societies........ 438
Medical Society of South Carolina........ 79
Medicine by galvanism........ 351
Medico-chirurgical Association........ 131
Medico-chirurgical Society........ 77
Membranous dysmenorrhœa........ 469
Memoranda for the disinfection of yellow fever 380
Meningitis, tubercular in the adult........ 60
Menorrhagia, viburnum prunifolium in........ 47
Menstrual disorders, Buffalo lithia waters in... 255
Menstruation, bromide of ammonium in........ 270
Metalloscopy and metallotherapy........ 18
Meteorology for 1878........ 152
Metric system........ 95
Metrorrhagia, plugging the cervix uteri for..... 464
Mercury, a cure for purpura hemorrhagica...... 14
Microphone, anticipation of........ 432
Milk abscess, arrest of........ 140
Milk diet........ 110
Milk diet in acute rheumatism........ 316
Milk diet in cystitis,........ 98
Milk test........ 392
Miscellanæ........ 461
Mistura bronchi........ 236
Misturæ........ 314
Mixture, a new........ 413
Modified Dover's powder........ 356
Modus operandi of skin grafting........ 21
Moles, removal of........ 235
Monobromated camphor in cholera infantum. 236
Monobromide of camphor as a hypnotic........ 28
Moon, new crater in the........ 191
Morbus coxarius, new treatment........ 103
Morphia hypodermically—caution........ 182
Morphia, hypodermic use of........ 406

Morphia, hypodermical use of, as a supporter of the heart's action.......... 325
Morphia mania.......... 23
Mound builders.......... 431
Mouth breathing, bronchitis from.......... 218
Mouth, nursing sore.......... 396
Muriate of pilocarpin in singultus.......... 150
Myrica and subnitrate of bismuth.......... 30

N

Nævi, tattooing of.......... 426, 429
Nævus, croton oil in.......... 146
Nævus, sodium ethylate in.......... 217
Narcosis, ice in the rectum in.......... 117
Narcotism from nutmeg.......... 344
Nasal catarrh, liquor bismuth for.......... 106
Nasal douche, insufflation powders against.......... 105
Nasal polypi.......... 461
Nasal polypus, carbolic acid in.......... 229
National board of health.......... 237
Nature, perfection of.......... 71
Nebulæ, disappearance of.......... 152
Neuralgia.......... 36, 47, 49
Neuralgia, Esmarch's tourniquet in.......... 386
Neuralgia of the fifth nerve, ammoniacal sulphate of copper in.......... 416
Neurose of the heart.......... 107
Niagara, stoppage of the falls of.......... 111
Nickel.......... 312
Nipples, cracked.......... 105
Nipples, suberine in excoriated.......... 267
Nitrite of amyl.......... 18
Nitrite of amyl in infantile convulsions.......... 106
Nitrogen, oxide of.......... 392
Nocturnal enuresis.......... 100
Nocturnal seminal emissions.......... 339
Nose, hemorrharge frcm the.......... 396
Notes from practice.......... 99
Nourishment in typhoid fever.......... 344
Nursing sore mouth.......... 396
Nux vomica.......... 18

O

Obstetric forceps.......... 108
Obstetrics and obstetrical surgery.......... 20
Obstetrics, chloral hydrate in.......... 88
Obstetrics, quinia in.......... 16
Œsophageal sound in hiccough.......... 310
Oil, chaulmoogra.......... 354
Oil, inhalation of Eucalyptus.......... 355
Oil in skin diseases.......... 263
Oil, petrolina.......... 432
Ointment, boracic acid.......... 156
Ointment, mild zinc.......... 193
Oleate of zinc.......... 109
Onychia scrofulosa.......... 104
Opening the mastoid process by surgical procedure.......... 259
Operation for phimosis.......... 228
Ophthalmia, ergotin in.......... 230
Ophthalmia, ergotin in acute.......... 144
Ophthalmia neonatorum.......... 434
Ophthalmia, purulent, of infants.......... 149
Opium, alkaloids of.......... 58
Opium, danger of, in chronic bright's disease.......... 109
Opium habit.......... 269. 270
Opium habit cured by galvanism.......... 332
Opium in peritonitis.......... 55
Opium, quinia with.......... 276
Opium smoking.......... 192
Origin of plants.......... 432
Otorrhœa.......... 122
Ovariotomy.......... 421, 458
Ovariotomy superseded.......... 227
Oxalate of cerium in chronic cough.......... 59
Oxalate of cerium in vomiting of pregnancy.......... 155
Oxalate of cerium in whooping-cough.......... 305
Oxide of nitrogen.......... 392
Oxide of zinc in diarrhœa.......... 223
Oxytocic, quinine a dangerous.......... 70
Oxygen in albuminuria.......... 430
Ozena, new treatment of.......... 427

P

Painful diarrhœa.......... 394
Pain, relief of, from the application of sulphate of copper.......... 228
Palatable castor oil.......... 313
Paracotoin in epidemic cholera.......... 150
Paralysis, infantile.......... 223
Parasite, a troublesome.......... 260
Pavor nocturnus.......... 475
Peach kernels for clearing water.......... 310
Peach leaves an anti-emetic.......... 76
Pencil, a voltaic.......... 271
Pepsin in dysentery.......... 234
Perchloride of iron as a topical application for chancre.......... 35
Perfection of nature.......... 71
Peristaltic persuaders, Dr. Kitchner's.......... 194
Peritonitis, opium in.......... 55
Permanganate of potash.......... 57
Pertussis.......... 396
Pertussis, dextro-quinine in.......... 417
Petrolina and petrolina oil.......... 432
Perineum, laceration of the.......... 457
Perimetritis.......... 460
Pharynx, fish-bones in.......... 224
Philippium, new metal.......... 232
Phosphate of lime.......... 430
Phosphide of zinc.......... 110, 268
Phosphorus, with malaria, the cause of yellow fever.......... 57
Phrænum, ulceration of.......... 65
Phthisis, glycerine in.......... 430
Phthysis syphilitic.......... 275
Phymosis as a cause of rupture in children.......... 98
Phymosis, operation for.......... 228
Physicians of Vicksburg.......... 78
Physiological action of borax.......... 272
Phytolacca decandra in mastitis.......... 483
Piles, rules for injecting.......... 220
Pile tumors, injecting.......... 30
Pills, laxative and anodine.......... 115
Pills, laxative tonic.......... 195
Pilocarpin as an abortifacient.......... 130
Pilocarpin in albuminuria.......... 108
Pimply-face, acne.......... 97
Pityriasis of the.......... 113
Plague in Russia.......... 77
Plague, the.......... 143
Plants, origin of.......... 432
Pleasant remedy for tooth ache.......... 262
Pleura, evacuation of pus from.......... 67
Pleurodynia.......... 114
Pneumonia.......... 273
Pneumonia, acetate of potassium in.......... 109
Pneumonia, aconite in.......... 404
Pneumonia, croupous.......... 201
Pneumonia interstitial.......... 388
Pneumonia, typhoid.......... 41, 81
Podophyllin.......... 34
Podophyllin, danger of.......... 462
Podophyllin, formula for administering.......... 473
Poison, antidote to.......... 265
Poisoning by carbolic acid.......... 29, 389
Poisoning by ice cream.......... 24
Poisoning by rhus.......... 306, 350
Poisoning by rhus toxicodendron.......... 154
Poisoning, tobacco.......... 321
Polypi nasal.......... 461
Polypi of the ear.......... 67
Popp's anatherine dentifrice.......... 435
Population of the globe.......... 112
Post-mortem delivery.......... 137
Post nasal catarrh.......... 434
Post-partum hemorrhage.......... 116
Potash, permanganate of.......... 57
Potassium, external application of the bromide of.......... 386
Potato eating and diphtheria,.......... 187, 315
Pott's disease.......... 12
Practical notes.......... 476
Practice, notes from.......... 99
Pregnancy. gingivitis in.......... 66
Pregnancy, graafian vesicle during.......... 107
Pregnancy in the elephant.......... 151
Pregnancy, salivation of.......... 264
Pregnant sickness, oxalate of cerium in.......... 176

Pregnant state, possible relations of tooth extraction to the........ 9
Premature labor in puerperal eclampsia........ 384
Preventing mammary abscess........ 375
Prickly heat, liquor arsenicalis in........ 195
Princely trial of an old remedy........ 271
Professional fraternity........ 196
Prolapse of the rectum in infants........ 187
Prophylaxis in yellow fever........ 300
Prophylaxis of puerperal convulsions........ 212
Propylamine chloride........ 261
Protoxide of nitrogen for producing prolonged insensibility........ 294
Pruritus ani........ 418
Pruritus vulvæ........ 113
Prussian blue in malarial fever........ 141
Psoriasis palmaris, eczema of the hand often miscalled........ 334
Psoriasis, pyrogallic acid in........ 230
Ptyalism........ 436
Public health, department of........ 78
Puerperal convulsions........ 30
Puerperal convulsions, jaborandi in........ 97
Puerperal convulsions, prophylaxis of........ 212
Puerperal eclampsia........ 94, 262
Puerperal fever........ 458
Pulmonary alterative pills........ 74
Purgatives, choice of........ 389
Purpura hemorrhagica........ 14
Pus evacuated from the pleura by inversion of the body........ 67
Putty, to remove dry........ 195
Pyrogallic acid in internal hemorrhage........ 265
Pyrogallic acid in psoriasis........ 230

Q

Quebracho, a new remedy in dyspnœa........ 309
Query........ 273
Quinia, capsicum with........ 195
Quinia in surgery and obstetrics........ 16
Quinia, sulphate-thymate of........ 104
Quinia with opium........ 276
Quinine, a dangerous oxytocic........ 70
Quinine, duty on........ 438
Quinine, to hasten the action of........ 350
Quinine, zinc against........ 296
Quinsy, simple treatment of........ 107

R

Rag weed as a remedy for rhus poisoning........ 469
Rapidity of thought in dreaming........ 231
Rectum, fissure of the........ 460
Rectum, removal of........ 19
Relief for dyspepsia........ [illegible]5
Remedies, new California........ 77
Removal of moles........ 235
Removal of the spleen........ 19
Report of some cases from the eye and ear and throat clinic........ 122
Reports, society........ 288
Resection of the infra-orbital nerve........ 127
Respiration, artificial........ 18
Resuscitation of a young girl apparently dead 290
Retention of urine, chloral in........ 225
Review of the past year........ 17
Rheumatic fever, acetate of potass. in........ 109
Rheumatic iritis........ 394
Rheumatism........ 144
Rheumatism, benzoic acid in........ 68
Rheumatism, buckeye in........ 469
Rheumatism, chronic........ 173
Rheumatism in scrofulous subjects........ 36
Rheumatism, milk diet in acute........ 316
Rheumatism of the diaphragm........ 221
Rheumatism, salicylate of soda in........ 190
Rheumatism, treatment of acute........ 145
Rhubarb and gentian draught........ 193
Rhus aromatica........ 462
Rhus poisoning........ 306, 350
Rhus toxicodendron, poisoning by........ 154
Ring-worm........ 72
Rosaniline in albuminuria........ 105
Rules for injecting piles........ 220
Rupture in children, phymosis as a cause of........ 98
Russia spiri........ 436
Russia, the plague in........ 77

S

Saint Benoit twins........ 31
Salicylate of quinine as an antipyretic........ 220
Salicylate of soda in rheumatism with high fever........ 190
Salicylic acid........ 227
Salicylic acid as an antiscorbutic........ 396
Salicylic acid as an antiseptic and antipyretic 66
Salicylic acid, concentrated solution of........ 354
Salicylic acid enemata in dysentery........ 190, 268
Salicylic acid in ear diseases........ 394
Salicylic acid in scarlet fever and diphtheria........ [illegible]
Salicylic acid mixture........ 236
Salicylic acid, solvent for........ 235
Salivation of pregnancy........ 264
Salve, lip........ 435
Sand blast........ 351
Sanitas, the new disinfectant........ 468
Scabies........ 368
Scale, new........ 312
Scarlatina, treatment of........ 161
Scarlatina, veratrum viride in........ 213
Scarlet fever, Berlin treatment of........ 146
Scarlet fever, chlorine mixture in........ 185
Scarlet fever, enlarged glands in........ 144
Scarlet fever, incubative period of........ 26
Scarlet fever, salicylic acid in........ 33
Scarlet fever, sulphurous acid in........ 108
Scars, how to avoid having........ 64
Sciatica, cure of........ 49
Sciatica, remedy for........ 224
Science benevolent? is........ 191
Scientific hoax........ 272
Scilline........ 385
Scleroderma........ 370
Scrofula, asclepias in........ 114
Scrofuloderma........ 370
Scrofulosa, onychia........ 104
Scrofulous subjects, rheumatism in........ 36
Seborrhœa........ 371
Secretion, to restrain excessive........ 194
Section, cæsarean........ 411
Sedative, codeia as a........ 297
Self-generating disinfectant........ 1[illegible]5
Seminal emissions; nocturnal........ 339
Sense of taste, the tongue and the........ 3[illegible]2
Septic organism, the thermal death point........ 32
Sexual hypochondriasis........ 361
Sexuality, fecundity and........ 352
Shock........ 76
Shock, a new treatment of........ 1[illegible]0
Short forceps superseded........ 308
Singultus cured by muriate of pilocarpin........ 150
Singultus, therapeutici of........ 343
Skin affections, bismuth in........ 225
Skin diseases........ 313
Skin diseases, linseed and oil in........ 263
Skin disease, vaseline in........ 221
Skin grafting, modus operandi........ 21
Skin irritation by administration of drugs........ 427
Skin, local treatment of diseases of the........ 368
Sleeplessness caused by cold feet........ 67
Sleeplessness, electricity for........ 65
Smoking opium........ 192
Sodium bromide in epilepsy........ 228
Sodium ethylate in nævus........ 217
Sodium, ethylate of........ 348
Solar systems other than our own........ 391
Solvent for salicylic acid........ 235
Sore mouth, nursing........ 396
South Carolina, Medical Society of........ 79
Southern Medical College........ 77, 158, 237, 279, 397
Spermatorrhœa........ 76
Sphygmophone........ 272
Sphymograph........ 312
Spinal curvatures........ 219
Spinal wounds........ 241
Spirits of nitre poisonous........ 74
Spleen, removal of........ 19
Squill, active principle of........ 385
Stigmata of maize in diseases of the bladder........ 414
Stomach, codeia for cancer of........ 70
Stomach, foreign body in the........ 428
Strangulated and incarcerated hernia, effect of posture in........ 257
Strangury from blisters prevented........ 266

Struggle for existence... 112
Strychnia as a tonic... 180
Subcutaneous injections of ergot in neuralgia.. 388
Suberine in excoriated nipples... 267
Subnitrate of bismuth... 30, 67
Subnitrate of bismuth in cholera infantum... 234
Substitute for the horse... 271
Sudden stoppage of hypodermic morphia... 209
Suggestions for treating swollen fingers... 36
Sulphate of cinchonia... 7
Sulphate of copper, relief of pain from the application of... 228
Sulphate of magnesia in dysentery... 234
Sulphate, thymate of... 104
Sulphuric acid in cholera... 475
Sulphuric ether in lumbago... 147
Sulphurous acid in scarlet fever... 108
Sulphurous acid, trichinæ destroyed by... 192
Sun spots, influence of... 112
Sunstroke... 292
Sun, where the, jumps a day... 111
Suppression of urine, Buffalo lithia waters in... 255
Suppression of urine, digitalis in... 190
Supra-pubic lithony... 29
Surgery... 18
Surgery, quinia in... 16
Surgery, obstetrics and obstetrical... 20
Surgical diseases, use of carbolic acid in... 28
Surgical Society, American... 237
Sweats, night... 17
Swollen fingers, suggestions for treating... 36
Sycosis... 474
Syphilderma... 475
Syphilis... 62
Syphilis, cases of... 304
Syphilis, contagium of... 349
Syphilis on the cure of wounds, influence of... 347
Syphilitic laryngitis... 68
Syphilitic neuralgia... 430
Syphilis, congenital... 459
Syphylitic phthisis... 175
Syphilitic skin diseases... 195
Syphilitic sores treated by immersion... 341
Syrup of chloral... 75

T

Tape-worm in cucumber... 68
Tape-worm, treatment of,... 103
Tattooing of Nævi...426, 429
Telemachon... 31
Telephonic phenomenon... 471
Telescope, a costly... 71
Test for arsenic... 72
Test for albumen... 316
Test, milk... 392
Tetanus... 66
Tetanus and chloroform... 110
Tetanus following ligation of hemorrhoids... 146
Tetanus from spinal wound... 241
Theory of electricity, a new... 232
Therapeutic effects of bryony... 268
Therapeutic effects of the subcutaneous injection of iron, condurango, etc... 420
Therapeutics, electro... 373
Therapeutics of singultus... 343
Therapeutic value of croton chloral... 412
Thermal death point of septic organism... 32
Thermometer in abdominal affections, clinical value of the... 188
Thermometer, urine... 390
Thermoscope and hydroscope... 208
Thunder, cause of... 391
Thymol... 20
Thymol, glycerole of... 435
Tinctura saponis viridis... 76
Tincture of iodine, Churchill's... 473
Tincture of iodine in cholera infantum... 276
Tincture of Myrrh in whooping cough... 108
Tincture of veratrum... 70
Tobacco, its injurious effects on the eye... 64
Toe nail, inverted... 264
Tongue and the sense of taste... 312
Tongue, condition of, valuable in the diagnosis of gastric disorders... 129
Tongue in diseases, appearance of the... 378
Tongue, tubercular ulcer of the... 219
Tonic, strychnia as a... 180
Tonsils, lemon juice for hypertrophied... 350
Tonsils, massage of the... 309
Tooth abscess opening into the antrum... 395
Toothache... 235
Toothache drops... 34
Toothache, pleasant remedy for... 262
Toothache remedy... 195
Tooth extraction and its relations to the pregnant state... 9
Tourniquet, Efmarch's, in neuralgia... 386
Tracheotomy with the thermo-cautery... 216
Transfusion... 17
Transplantation of a dog's cornea to the human eye... 347
Travel, rules for sending consumptives to... 98
Treatment of cardiac dyspnœa... 424
Treatment of chronic trigeminal neuralgia... 47
Treatment of deep sinuses by Villate's mixture... 33
Treatment of puerperal convulsions... 30
Treatment of the drowned... 447
Trichinæ destroyed by sulphurous acid... 192
Trichinæ in the flesh of geese... 156
Tubercular ulcer of the tongue... 219
Tumor, extraction of encapsuled placental... 284
Tumor injected with morphia before extirpation... 106
Turpentine vapor baths... 67
Twins, St. Benoit... 31
Typhoid fever after forty can one have?... 12[illegible]
Typhoid fever, baptisia tinctoria in... 306
Typhus, contagiousness of abdominal... 350
Typoid fever during infancy... 308
Typhoid fever, nourishment in... 344

U

Ulceration of the phrænum... 65
Ulcer Nushbaum... 62
Ulcer of the tongue... 219
Ulcers and abrasions... 436
Ulcers, Martin's bandage in chronic... 381
Ulcers of the cornea, phlyctenular... 307
Ulcers, treatment of... 70
Unhealthy local and syphilitical sores, treated by immersion... 341
Uranine... 32
Urethra, carbuncle of the... 457
Uric acid diathesis... 109
Uric acid diathesis, Buffalo lithia waters in... 255
Urine, chloral in retention of... 225
Urine, digitalis in suppression of... 190
Urine, Grisolle's pills for incontinence of... 116
Urine, hypodermic injections of tincture of ergot for retention of... 136
Urine icteric... 110
Urine in children, incontinence of... 388
Urine thermometer... 390
Urticaria caused by sulphate of cinchonia... 147
Uses of jaborandi... 25
Uterine applications... 425
Uterine hemorrhage, warm fomentations in... 148
Uterine injections, cause of death in... 348
Uterus, anteflexion of the... 433
Uterus, sclerotic acid in fibroid tumors of the.. 70

V

Vaginal applications... 425
Vaginismus... 113
Vaginitis... 116
Vaseline and unguentum vaselini plumbicum in skin disease... 221
Vaccina developed after fourteen years incubation of the virus... 253
Vaccination as a preventive of other diseases than variola... 268
Vagina in labor, warm water into the... 301
Valuable if true... 472
Value of the hypophosphites... 337
Veratrum, tincture of... 70
Veratrum viride in scarlatina... 213
Vick's floral guide... 39
Viburnum prunifolium for controlling the
Viburnum prunifolium... 125, 273
Villate's mixture, treatment of sinuses by... 33

Viburnum prunifolium in threatened abortion and in menorrhagia... 47
Vinegar fumes in bronchitis... 66
Vicksburg, physicians of... 78
Vomica, nux... 18
Vomiting, spirit of walnut for... 114
Vomiting of pregnancy... 163
Vomiting, interesting case of... 170
Vomiting, iodide of potassium in... 269
Voltaic pencil... 271
Vomiting of pregnancy, oxalate of cerium in.. 155
Vulcanized india rubber in blepharitis... 108
Vulcanized rubber nipples, dangers of... 236
Vulvæ pruritis... 113

W

Walnut for vomiting, spirit of... 114
Warm water into the vagina in labor... 301
Whirlwinds... 72
Whiskey hypodermically in collapse from loss of blood... 164
Whooping-cough... 65
Whooping-cough, bryonia and drosera in 110, 266
Whooping-cough, cubebs in... 476
Whooping-cough, jaborandi in... 215
Whooping-cough, oxalate of cerium in... 305
Whooping-cough, picrate of ammonia in... 101
Whooping-cough, tincture of mirrh in... 108
Wine of ipecac in vomiting of pregnancy... 163
Womb, anteflexion of... 105
Womb, bromine injections in cancer of the.... 270
World? how old is the... 311
Worm kidney... 71
Worms... 233
Wounds, dry dressing in... 108
Wounds... 109
Wound dressing, carbolized jute as... 174
Wounds, influence of syphilis on the cure of... 347
Wound to the brain... 366
Wounds, dressing head... 376

Y

Yellow fever, phosphorus the cause of... 57
Yellow fever rooms, Prof. Jones' experiments with the water and air from the... 181
Yellow fever, cost of... 192
Yellow fever, Buffalo Lithia Waters in... 255
Yellow fever, prophylaxis in... 300
Yellow fever, memoranda for the disinfection of... 380
Yerba santa in catarrhal affections... 476

Z

Zinc, oleate of... 109
Zinc ointment... 193
Zinc, oxide of... 193
Zinc, phosphide of... 268
Zinc against quinine... 296

### INDEX OF AUTHORS.

AMES, W. N.—Whisky hypodermically in collapse from loss of blood... 165
ANDERSON, R. B.—Childbirth without pain, 86
ANDERSON, S. H.—Report of a case of locomotor attaxie progressive, with some unusual complications... 5
Is croupus pneumonia a specific infectious disease?... 327
BOUCHELLE, L. B.—Hydrate of chloral in obstetrics... 88
CALHOUN, A. W.—Report of some cases from the eye and ear and throat clinic... 121
Tobacco poisoning and its effect upon the eyesight... 321
CARPENTER, W. W.—Diphtheria... 209
It steps down from its airy throne... 328
GLAZENER, G. S.—Viburnum prunifolium... 125
GREENLEY, T. R.—Is consumption contagious?... 1
On the treatment of scarlatina... 161
On the hypodermic use of morphia as a supporter of the heart's action... 325
HOBBS, A. G—Croupus pneumonia a specific infectious disease... 201
An encapsuled placental tumor extracted from the uterus five weeks after parturition... 284
Aborted croupus pneumonia... 401
HUNT, W. D.—Foreign body in the ear... 9
JOHNSON, LINDSAY.—Vaccina developed after fourteen years incubation of the virus... 253
Enlargement of the testis in child two years of age... 403
KINNE, A. F.—The continuity theory in diphtheria and the practice of test abrasions.... 281
LALLERSTEDT, T. S.—Sulphate of cinchonia 7
Cinchonia alkaloid... 207
LAWSON, H. M.—Gonorrhœa, how to cure it easily and quickly... 290
Gonorrhœa... 374
NISBET, D. B.—Viburnum prunifolium in threatened abortion and in menorrhagia... 47
Vomiting of pregnancy—treatment with wine of ipecac... 163
PAYNE, ALB[illegible]N S.—Typhoid pneumonia...41 81
POWELL, T. S.—Introductory address upon southern institutions... 441
RUSHING, F. M.—A case of empyema or suppurative pleuritis... 286
SUGGS, I. J.—Treatment of fistula in ano a new method... 89
TADLOCK, A. B.—Spinal wound—tetanus—death and post-mortem... 241
WELLS, E. F.—Injuries of the head—bloodletting in extravasation... 123

### *BOOK NOTICES.*

RECEIPTED.—H. T. Chevis, '78 & '79; W. W. Leak, '78; W. E. Broute, '78; T. Courtney, '79; J. W. Lee, '79; G. W. Bowling, April '79; J. Alexander, '78; O. L. Alexander, '78; H. J. Hester, '79; Mrs. Albert Smith, '79; Robt. James, '79; John A. Gordon, '78; A. T. Park, '79; P. W. Callahan, '79; A. W. Calhoun, July '78; T. A. Boggain, '78; R. J. Talbert, '79; T. L. H. Cook, '79; H. H. Bate, '78; S. E. Bratton, '78; C. Bethune, '78; J. J. Purcell, '79; G. L. Doster, '79; H. Kelley, 79; T. J. Brasher' 79; G. R. Dozier, '78; T. P. Oliver, '79; C. C. Hammond, '79; N. G. West, '79; R. H. Edwards, '79; R. J. Mathews, '79; R. C. Wily, '78; W. M. Garrard, '78; W. J. Lee, '79; J. E. Terrell, '79; E. S. Foster, '78; John Lee, 6m's '79; J. W. Unger, '79; D. B. Searcy, '79; J. W. Bailey, '79; M. R. Deadwyler, '78-79; W. T. Mathews, '79; A. P. Harris, '79; L. W. Mobley, to Aug. '79; J. F. Price, '79; S. W. Eaton, '79; C. H. Jones, '79; T. M. Palmer.

